# Linking Mediterranean Diet and Lifestyle with Cardio Metabolic Disease and Depressive Symptoms: A Study on the Elderly in Europe

**DOI:** 10.3390/ijerph17197053

**Published:** 2020-09-26

**Authors:** Judit Vall Castelló, Charisse Tubianosa

**Affiliations:** 1 Department of Economics, Universitat de Barcelona, 08034 Barcelona, Spain; 2 Barcelona Institute of Economics, Universitat de Barcelona, 08034 Barcelona, Spain; charisse.tubianosa@ub.edu

**Keywords:** aging, dietary patterns, cardio metabolic disease, physical activity, Mediterranean diet

## Abstract

Against a backdrop of an aging population in Europe, promoting health in older adults becomes a pressing issue. This study aimed to explore if correlations exist between the adherence to the Mediterranean diet and specific health outcomes such as the incidence of chronic cardio metabolic illnesses and experiencing depressive symptoms for elderly individuals. We also looked into probable links between regularly engaging in vigorous physical activities and these health outcomes. Our goal was to clearly demonstrate these relationships while controlling for several individual characteristics and socio-demographic factors on a cross-national scale within Europe. Using the Survey of Health, Aging and Retirement in Europe (SHARE) data for adults aged 50 years and above, we found that following the Mediterranean diet was negatively correlated with the incidence of chronic illnesses, as well as with levels of depressive symptoms. These results were robust to the inclusion of a number of individual and socio-demographic controls. We also showed that regular participation in sports and other strenuous physical activities were associated with lesser chronic disorders and lower levels of depressive symptoms. These findings may have important implications in formulating preventive interventions on ensuring the quality of life of the older population.

## 1. Introduction

The combination of a rapidly aging world population and increased life expectancy bring with it a complexity of concerns. Foremost among them, promoting health in older adults becomes a pressing issue. The prediction is that by the year 2050, about “one in every four persons in Europe and Northern America could be aged 65 years or over” [1] (p. 18). According to the European Commission’s 2018 Aging Report, “the old-age dependency ratio (the ratio of inactive persons aged 65 and above over the employed working-age population of 20−64) in the EU is forecast to increase from 43.1% in 2016 to 68.5% in 2070” [2] (p. 55). Aging has often been characterized by increasing susceptibility to the development of chronic illnesses, consequently to a rising risk of multimorbidity, as well as a tendency for geriatric obesity [3,4,5,6]. Multimorbidity is defined as “having multiple chronic conditions at the same time” [7] (p. 7), and the accelerating incidence of which will have significant implications not only for the individuals themselves, but also for healthcare systems and national economies [8]. Given this, it is in everyone’s interest to ensure resilience toward the health and well-being among the older population.

There is abundant literature linking the importance between nutrition, diet, and lifestyle with the quality of life and health of older adults [9,10]. The Mediterranean diet, for instance, has often been considered a high quality diet. Several studies [11,12,13] have highlighted the benefits of following a Mediterranean diet, particularly for those suffering from chronic conditions related to cardio metabolic disorders (CMD), particularly among older adults. In addition, this diet has even been advocated to mitigate risks related to depressive symptoms [14]. There is also growing evidence on increased and sustained physical activity being positively linked with healthy aging and life satisfaction [15,16,17,18].

Our study centers on this area of research by revisiting how the Mediterranean diet and physical activity link with specific health indicators of older adults with some novelties. Understanding the aging process can be a challenging endeavor given the myriad of factors that may be involved. Many studies tend to focus solely on diets, nutrition and health outcomes, though a few of them also control for socio-demographic aspects and consider lifestyle behaviors [19,20]. However, most of them are limited to national, cross-section studies, or case studies with small sample sizes. We contribute by exploring whether these links continue to be significant even when adjusting for several potential confounding factors related to personal and socio-demographic characteristics. Aside from including a large number of controls, our study adds to the previous literature with its broader cross-national scope and a considerably larger set of panel observations. Specifically, using a large longitudinal database encompassing 27 European countries, the main objectives of our study are to: (1) test if correlations exist between adhering to the Mediterranean diet and variations in health outcomes, such as the incidence of chronic CMDs, the levels of body-mass index (BMI) and the levels of depressive symptoms; (2) verify if correlations can also be observed between a physically active and healthy lifestyle with the same health outcomes; and, should these associations prove to be significant, evaluate the strength or intensity of these relationships with the inclusion of control variables.

## 2. Materials and Methods

### 2.1. Data Description, Key Variables, Covariates and Measurement

We use the Survey of Health, Aging and Retirement in Europe (SHARE) panel database. The survey design has been documented separately [21,22]. This is a rich and large database, which provides information not only on socio-demographics (e.g., gender, age, marital status, education level, ethnicity, household income) and physical health (e.g., BMI, chronic illnesses), but also on behavioral health risks (e.g., dietary patterns, physical activity, smoking habits) and mental well-being (e.g., level of depressive symptoms, perceived quality of life). These data allow us to look at these multifaceted dimensions, following respondents at different points in time. In its latest form, SHARE has data from the years 2004 to 2017 featured in 7 waves. Because of this, we can include controls for individual characteristics so as to “net out” the effects of individual factors on the outcome measures analyzed. With SHARE, we can also exploit cross-national variation since it includes respondents from various European countries, enabling cross-country and cross-wave comparability. For this study, we make use of waves 4, 5, 6 and 7 (surveys taken from the years 2011 to 2017) which are the periods with data on dietary patterns. The number of observations ranges from 126,729 to 224,364 depending on the variables considered. The variable related to chronic illnesses has the largest number of observations, while those pertaining to diet have the smallest. The variable reflecting information on depressive symptoms is based on a sample of 167,698, while for BMI we have 218,151 observations. The 27 countries included in the sample are: Austria, Germany, Sweden, Netherlands, Spain, Italy, France, Denmark, Greece, Switzerland, Belgium, Czech Republic, Poland, Luxembourg, Hungary, Portugal, Slovenia, Cyprus, Estonia, Croatia, Lithuania, Bulgaria, Finland, Latvia, Malta, Romania and Slovakia. Taking the original sample from waves 4 to 7, respondents were aged 50 to 112 years old. However, in the analysis we put a restriction on the age up to 80, as will be explained in Section 2.2. The resulting mean age in our sample is 65. The most frequent age categories are 55−59, 60−64 and 65−69. From the sample, 56% are female. The succeeding subsections describe the groups of variables we use for the analysis.

#### 2.1.1. Health Outcome Indicators

We are interested in the indicators related to CMDs, the level of BMI and mental well-being. SHARE covers several types of diseases. For this study, we generated a composite chronic illness variable that considers only those illnesses related to cardiovascular diseases and Type 2 diabetes—all belonging to CMDs. Specifically, we included: heart attack and other heart problems, hypertension, high blood cholesterol, stroke or cerebral vascular disease and diabetes or high blood sugar. For this variable, a higher value indicates the individual suffers more CMDs. A value of 5 means this respondent reported suffering from all the five types of CMDs included in the survey. We use the variable for BMI provided by SHARE which has been calculated from individual weight and height, and has continuous values. Values equal and greater than 25 indicate being overweight or obese (≥30).

For mental well-being, we make use of the EURO-D scale included in the SHARE questionnaire. This is a composite index which is a symptom scale measuring the level of depressive symptoms with values ranging from 0 (having no symptoms) to 12: “The scale includes 12 items that are summed and clinically significant depression is defined as a score greater than 3. This cut-point has been validated in the EURODEP study against a variety of clinically relevant indicators. Respondents scoring above this level would likely be diagnosed as suffering from a depressive disorder, for which therapeutic intervention would be indicated” [23] (p. 5). A tabulation of how many observations have been classified under the incidence of CMD, BMI (in broad groupings) and the scales of EURO-D are in Table A1, Table A2 and Table A3. From our sample, about 56% are indicated to be suffering one CMD or more, 65% are classified as between being overweight and obese, while 26% have reported to be depressive symptomatic.

#### 2.1.2. Mediterranean Diet

Data on dietary patterns had been assessed through the following survey questions:

1. In a regular week, how often do you have a serving of dairy products, such as a glass of milk, cheese in a sandwich, a cup of yogurt or a can of high protein supplement?”
2. In a regular week, how often do you have a serving of legumes, beans or eggs?”
3. In a regular week, how often do you eat meat, fish or poultry?”
4. In a regular week, how often do you consume a serving of fruits or vegetables?”

The potential answers were (1) Less than once a week; (2) Once a week; (3) Twice a week; (4) 3–6 times a week; (5) Every day. A summary of how many individuals chose each response category can be found in Table A4. From this data, we constructed a binary index identifying which of the respondents in our sample may be classified as subscribing to a Mediterranean diet (value = 1 if following the diet; 0 if otherwise). In this study, we loosely followed the definition of the Mediterranean diet from earlier studies [24,25] as the daily consumption of fruits or vegetables; and frequent intake of legumes, beans or eggs and meat, fish or poultry. Although the database does not allow us to separate red meat from the other types of meat, we later test if our results are robust to marginal changes in the definition of Mediterranean diet.

#### 2.1.3. Variables Related to Individual Socio-Demographic Characteristics

For the analysis, we take into account some individual characteristics available in SHARE, such as gender (female = 1; male = 0), age, marital status and ethnicity. Marital status was recoded as a binary indicator to reflect if the respondent lives alone (separated, divorced, single, or widowed; value = 1), or lives with a partner, whether married or not (value = 0). We also generated a dummy variable for ethnicity based on the respondent’s declared country of birth. We used the definitions from the US Census ethnicity classification (Hispanic = 1; Non-Hispanic = 0) [26].

#### 2.1.4. Other Personal Traits and Lifestyle Indicators

Apart from the characteristics described in 2.1.3, we also include variables related to education (based on the International Standard Classification of Education (ISCED) 1997). From this, we generated dummy variables for the highest education level achieved by the individual, whether tertiary, secondary, or primary/none. We also recoded the employment status variable from SHARE to reflect if the individual is employed (=1) or inactive/retired/unemployed (=0). Annual household income is measured in euros. Specific lifestyle indicators relate to physical activity and smoking behavior. We recoded the original SHARE variable on reported frequency of doing vigorous activities: sports, heavy housework, or involvement in a physically taxing job. The resulting variable indicates whether the individual regularly engages in sports/vigorous activities (=1) or hardly/not (=0). We use the SHARE variable that gives information if the respondent has ever smoked daily (no = 1; yes = 0).

### 2.2. Statistical Analysis

To test for the relationship between our various health indicators and the Mediterranean diet, lifestyle behaviors and variables related to individual and socio-demographic characteristics, we use a fixed effects model and utilize the software Stata^®^ 14.0 (Statacorp, College Station, TX, USA) to perform the regressions.

(1) $$Y_{ijt}=\alpha +\beta_{1}Med_{ijt}+\beta_{2}X_{ijt}+\beta_{3}LH_{ijt}+\beta_{4}X_{ijt}^{2}+\delta_{j}+\gamma_{t}+\epsilon_{ijt},$$

where $Y_{ijt}$ is one of the three health indicators we are considering (chronic CMD, BMI, or level of depressive symptoms) for the elderly respondent *i* interviewed in country *j* on year *t* (or wave *t*), $Med_{ijt}$ is a binary variable indicating whether the respondent adheres to the Mediterranean diet or not, $X_{ijt}$ is a vector of individual and socio-demographic characteristics, depending on the specification we are using (gender, age, marital status, ethnicity, education, income and employment status), $LH_{ijt}$ refers to lifestyle indicators (smoking habits and physical activity), $\delta_{j}$ and $\gamma_{t}$ are the country and year fixed effects. $X_{ijt}^{2}$ takes the quadratic form of age and of household income to account for the possibility of a non-linear relationship with these variables. $\beta_{1}$ measures the strength of the relationship between the particular health indicator and subscribing to the Mediterranean diet, while $\beta_{3}$ reflects the possible association between lifestyle behaviors and the health outcome. For our analyses, we employ three specifications: (1) includes only the Mediterranean diet variable ($Med_{ijt}$); (2) incorporates individual characteristics (i.e., gender, age, marital status and ethnicity) together with the diet variable; and (3) puts (1) and (2) together with the other socio-demographic and lifestyle dimensions (i.e., education, employment, income, physical activity and smoking habits). The fixed effects for the survey year (wave) and country should control for unobserved characteristics that can affect our health indicators associated with the survey year and the countries where the interviews were conducted. Standard errors are clustered at the country level. We restrict our sample to survey respondents aged 50 and above, while putting a cap at 80 years of age to sidestep possible selection bias that may arise. We also exclude data from Israel given our focus on European countries.

## 3. Results

Before going into the main regression results, we perform an exploratory analysis of the data. The descriptive statistics are in Table 1, showing the mean and standard deviations for the variables we use. Results from the statistical analysis are organized according to the health indicator of interest, from Section 3.1 to Section 3.3. Results from a robustness check are in Section 3.4.

**Figure 1:**
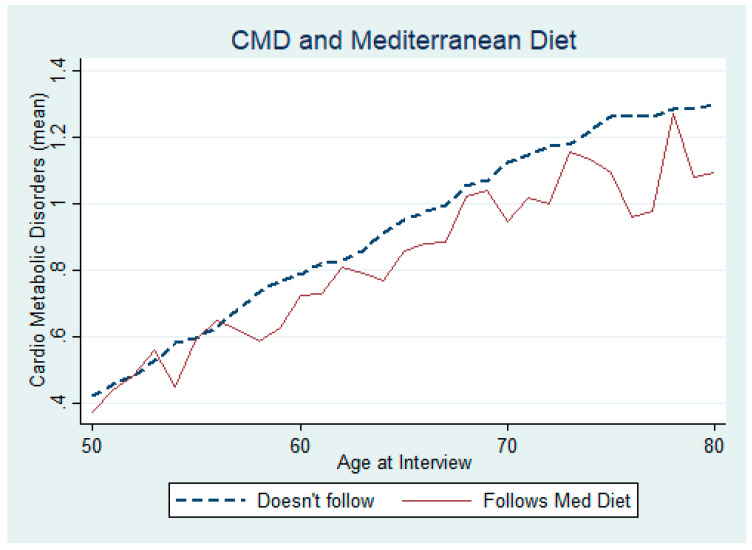
Incidence of chronic cardio metabolic disorders (CMDs) and following (or not following) the Mediterranean diet.

**Figure 2:**
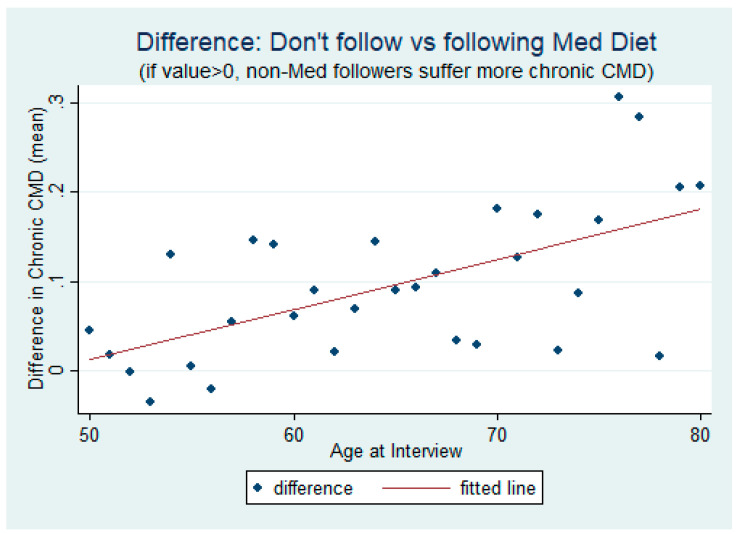
The difference in incidence of chronic CMDs between those following and not following the Mediterranean diet.

Prior to formally testing, we provide graphical overviews on how CMDs relate with the different variables we use in the study. In Figure 1, we compare the CMD incidence between those who adhere to the Mediterranean diet and those who do not, by age. The graph clearly indicates that, on average, there is a marked difference in CMD incidence between those following the diet and those who do not—generally showing a lower number of these chronic diseases for those who do subscribe to the diet. We also employ a scatter plot in Figure 2 to show the difference in CMD incidence between those who follow the Mediterranean diet and those who do not, again by age. The difference in CMDs is generally greater than zero between those who take the diet and those who do not. This difference increases with age, as depicted by the fitted line.

**Figure 3:**
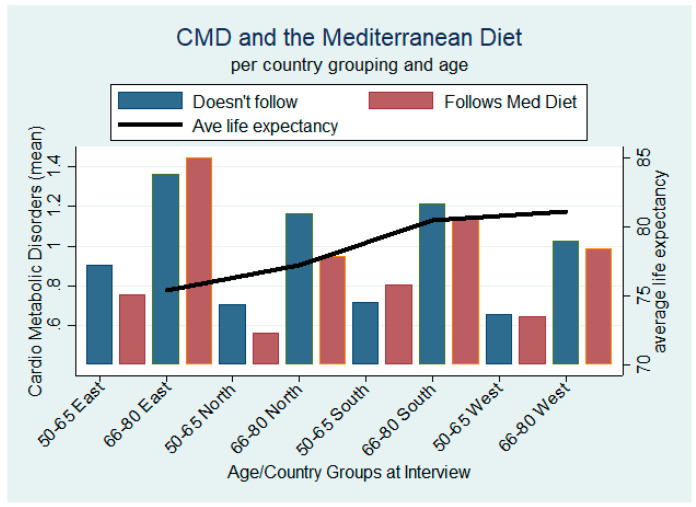
CMDs and the Mediterranean diet: by age and country groups.

Dividing our observations into country groupings using the UN geoscheme for Europe (details in Table A5) and into large age groupings (50−65 and 66−80), Figure 3 shows that individuals in every first age group (50−65) who follow the Mediterranean diet have lower CMDs in every country grouping, except those from the South. The black line across the graph indicates the average life expectancy for each country grouping and from which we note that countries in the East have the lowest at 75.4 years. For the higher age bracket, individuals who observe the diet are associated with a lower CMD incidence, except those from Eastern Europe.

**Figure 4:**
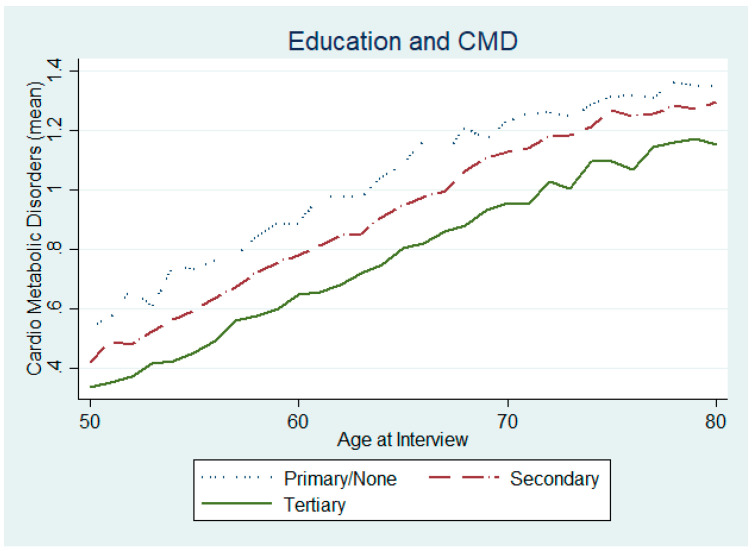
Chronic CMDs and level of education.

**Figure 5:**
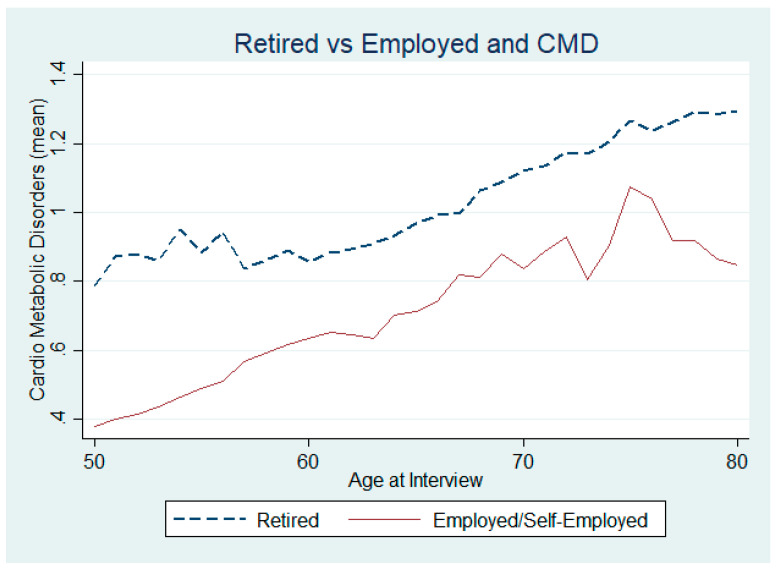
Chronic CMDs between employed and retired individuals.

Could there also be a difference in CMDs in terms of education and employment characteristics? From Figure 4, we can already infer that individuals with the highest level of education (solid green line) are also those with the lowest incidence of CMD, at any age. In terms of employment status, we also observe a difference between those who have identified as “Retired” (Figure 5) in the survey and those who continue to be employed or self-employed, with those in the latter group having a lower incidence of diseases.

### 3.1. Cardio Metabolic Disorders (CMDs)

Following the results presented in Table 2, in our baseline specification in column (1), we see a strong negative correlation with the Mediterranean diet, without adjusting for the other variables. The strength of the relationship holds when we include individual characteristics (column 2) and the full model including the socio-demographic and lifestyle variables (column 3). However, in column (3), the size of the coefficient for the Mediterranean diet decreases slightly. From this specification, the other variables which are highly correlated (*p* < 0.01) with CMD are gender, age, age^2, tertiary and secondary levels education (the education variable for primary/none is omitted due to collinearity), smoking, employment status and engagement in sports/vigorous physical activities. Ethnicity is also significant (*p* < 0.05).

### 3.2. Body-Mass Index (BMI)

For the results related with BMI (Table 3), we find no significant association relating with adherence to the Mediterranean diet for any of the specifications. We do find highly significant negative correlations with being female (columns 2 and 3), having more education, being engaged in sports and being employed (column 3). In fact, we observe that having tertiary level education (*p* < 0.01) has the strongest negative relationship with BMI in terms of magnitude, in comparison with all the other variables in column (3). The variables related to age are also highly significant, indicating that growing older is correlated with higher BMI, but only up to a certain point since the variable age^2 has a negative sign (both columns 2 and 3). We also note that respondents who have indicated that they do not/never have smoked daily seem to be associated with higher BMI levels. Ethnicity loses significance when adjusted for other variables in the third specification.

### 3.3. Depressive Symptoms

Table 4 presents the results related to depressive symptoms. We find a negative and significant (*p* < 0.05) relationship between following the Mediterranean diet and depressive symptoms, indicating that respondents who follow the diet are likely to report lower levels of depressive symptoms (column 1). Similar to the case with CMDs, the size of the correlation holds, with just a slight drop, even when adjusted for other characteristics (columns 2 and 3). However, differing from the results in Table 2, being female shows a positive and highly significant (*p* < 0.01) association with depressive symptoms (column 2), which remains significant even after the inclusion of other variables (column 3). Having no partner/living alone and being Hispanic are also observed to be positive and strongly significant in relation with depressive symptoms, though ethnicity is only significant at the 5% level once adjusted for the other variables (column 3). Education also seems to have a highly significant relationship (*p* < 0.01), indicating those with higher levels (tertiary) are associated with lower reported depressive symptoms. Engagement in sports, not having been habitual smokers, and being employed are also highly (*p* < 0.01) and negatively correlated with depressive symptom levels. As with the other health outcomes, the age variables are highly significant for specifications 2 and 3, with age having a negative coefficient, while that of age^2 is positive.

The household income variables are only significant in relation with depressive symptoms, though the magnitudes of the relationship are almost zero.

### 3.4. Robustness Check

As a robustness check, we calibrate the consumption frequency of the meat and the legumes components of our Mediterranean diet variable and run the analysis using specifications (1) and (3) described in Section 2.2. The results are in Table 5 where we see that the direction of the main results are unaffected by marginal changes in the definition of the Mediterranean diet.

## 4. Discussion

Our results confirmed a highly significant and negative relationship between the Mediterranean diet and two of our health outcomes. We saw that individuals following this diet were associated with a lower incidence of CMDs and lower levels of depressive symptoms. The magnitude of the association declined slightly when we adjusted for the covariates, but the significance remained stable (*p* < 0.05). These results follow in the tradition of previous studies that have found positive links between the Mediterranean diet and health in older adults [27,28,29], though ours has implications for Europe in general with the use of our broadly-scoped sample. The results were also robust when we calibrated the composition of our Mediterranean diet variable to slightly reduce the frequency of meats and legumes consumption, where we even observed a higher level of significance (*p* < 0.01) and magnitude in relation with the level of depressive symptoms. We note from the literature, however, that the age factor still leaves more to be explored in relation with mental well-being in old age [30,31], thus we focus only on the direction of the relationship from the results. For BMI, we found no significant links with the Mediterranean diet. We are mindful of the limitations of the usefulness of BMI as a measure of health [32,33] so additional information is likely called for to be able to provide a better analysis in relating this diet with BMI.

In line with our hypothesis, those who were more active and occupied, in terms of sports or employment, were shown to be less likely to have more CMDs, tended to have lower BMI, and reported to be less depressive symptomatic. This is consistent with studies mentioning the value of continued engagement in economic activities and more physical activity in having lesser cardiovascular problems, other chronic illnesses and overall better health [15,18,34,35,36,37]. In addition, apart from the healthy lifestyle habit of engaging in sports, not being a regular smoker was also linked with better health (i.e., lower CMDs and lower levels of depressive symptoms). However, we observed that the correlation between non-habitual smoking and BMI was positive. This is not an unusual result following previous studies showing that discontinuance of smoking tends to be associated with weight gain [38,39,40].

As previously mentioned, health and well-being in older adults are complex and multifaceted themes. Given this, it would be noteworthy to highlight the results for our covariates which generally conform with what has been shown in the literature, giving more credence to our analysis. This may also open avenues for future research. For instance, we found that being female was highly associated with lesser CMDs. Older women in our sample were less likely to have higher CMD incidence. Though there are studies which point out that “women may be underrepresented in research on heart disease” and likely misdiagnosed [41,42]. But when it came to mental well-being, our study indicated that being female tended to be correlated with higher levels of depressive symptoms. Previous studies in this area share the consensus that older women have an elevated risk for depressive symptoms, are increasingly diagnosed with depressive disorders and are less positive with regard to their perceived health in comparison with men [41,43,44,45,46]. In more stable standing, health literature has established the protective factor provided by education [47,48,49]. Our results confirmed this negative association with our health outcomes, where tertiary level education in particular showed larger magnitudes versus that for the secondary level. Being Hispanic was associated with worse health outcomes. This would be consistent with studies showing that mortality rates from diabetes tend to be higher for Hispanics than non-Hispanics, and a larger proportion with chronic illnesses [50,51,52]. Nonetheless, we note the limitation of our ethnicity variable before drawing any conclusions. The variable was generated based on the respondent’s declared country of birth, implying an imperfect measure to determine true ethnicity.

Lastly, the state of living with a partner or not (from marital status) seems to have an interesting relationship with health. We found that living apart from one’s partner (or being single, widowed) in old age was found to be strongly correlated with higher levels of reported depressive symptoms, similar to existing literature [53,54]. However, not living with a partner seemed to be linked with lower levels of BMI—consistent with some studies that show “transitions out of marriage are associated with weight loss” [55,56]. It may certainly be argued that the quality of the relationship is an important characteristic to take into account, although this aspect is outside the scope of our study. However, the main results draw attention to the importance of relationships relative to the quality of life [57] and could be compelling to explore in a future study.

## 5. Conclusions

The main findings of our study confirmed the direct association between adherence to the Mediterranean diet and a lower incidence of CMDs, and with lower levels of reported depressive symptoms among older adults. We also confirmed that regular vigorous physical activity and non-habitual smoking were associated with fewer CMDs and better mental well-being. We reached these results while accounting for the multidimensionality of these health outcomes—by controlling for several individual and socio-demographic characteristics. In addition, our study extends the current latitude of the literature given that the evaluation was within the context of a large longitudinal sample covering 27 European countries. These findings, having been situated in a broader geographic setting, emphasize the importance of formulating a holistic program when caring for the elderly, not least among them mindful attention to diet and nutrition.

## Figures and Tables

**Table 1:** Descriptive statistics of key variables ^1^.

	N	Mean	SD	Min	Max
Chronic CMD	224,364	0.90	1.01	0	5
BMI ^2^ (kg/m^2^)	218,151	27.12	4.71	12.46	98.62
EURO-D	167,698	2.37	2.22	0	12
Mediterranean diet (yes = 1)	126,729	0.07	0.25	0	1
Gender (female = 1)	224,364	0.56	0.50	0	1
Marital status (not living with partner = 1)	223,297	0.28	0.45	0	1
age (years)	224,364	64.99	7.79	50.00	80.00
Ethnicity (Hispanic = 1)	223,000	0.07	0.26	0	1
educ: tertiary (1/0)	221,367	0.22	0.42	0	1
educ: secondary (1/0)	221,367	0.59	0.49	0	1
educ: primary/none (1/0)	221,367	0.18	0.39	0	1
sports/physical activity (yes = 1)	171,906	0.60	0.49	0	1
ever smoked daily (no = 1)	170,073	0.52	0.50	0	1
household income (euros)	172,531	32,780.64	60,592.12	0	9,589,806.00
employment status (employed = 1)	219,663	0.29	0.45	0	1

^1^ SHARE data for waves 4, 5, 6 and 7 restricted to observations aged 50−80. ^2^ Note that there are 17 out of 218,151 observations with BMI > 70 (which represents only a 0.007% of our sample) and 89 observations with BMI < 15 (representing 0.041% of the sample).

**Table 2:** Regression analysis relating CMDs with the Mediterranean diet, individual and socio-demographic characteristics and lifestyle behaviors.

Covariates	Dependent Variable: Chronic_CMD (Incidence of CMD)		
	(1)	(2)	(3)
	CMD_Diet	CMD_Individual	CMD_All Vars
MED_diet (follows = 1; no = 0)	−0.0526 **	−0.0552 ***	−0.0456 **
	(0.0207)	(0.0202)	(0.0210)
female (female = 1; male = 0)		−0.0670 ***	−0.0862 ***
		(0.0236)	(0.0227)
marital status (NOT living with partner = 1; yes = 0)		0.0108	−0.0051
		(0.0087)	(0.0086)
age		0.0722 ***	0.0512 ***
		(0.0090)	(0.0084)
age^2		−0.0003 ***	−0.0002 ***
		(0.0001)	(0.0001)
ethnicity (Hispanic = 1; Non-Hisp = 0)		0.0930 *	0.1007 **
		(0.0491)	(0.0502)
tertiary educ (tertiary = 1; No = 0)			−0.2215 ***
			(0.0219)
secondary educ (secondary = 1: No = 0)			−0.1197 ***
			(0.0210)
sports (active sports = 1; Not = 0)			−0.1715 ***
			(0.0176)
ever smoked daily (no = 1; yes = 0)			−0.0537 ***
			(0.0126)
household income			−0.0000
			(0.0000)
household income^2			0.0000
			(0.0000)
employment status (employed = 1; no = 0)			−0.1303 ***
			(0.0129)
Constant	0.8479 ***	−2.4345 ***	−1.1856 ***
	(0.0056)	(0.3006)	(0.2968)
Observations	129,083	128,062	124,549
Number of id_n	85,836	85,150	83,600
Year FE	YES	YES	YES
COUNTRY FE	YES	YES	YES

Robust standard errors in parentheses. *** *p* < 0.01, ** *p* < 0.05, * *p* < 0.1.

**Table 3:** Regression analysis relating BMI with the Mediterranean diet, individual and socio-demographic characteristics and lifestyle behaviors.

Covariates	Dependent Variable: BMI (Kg/M^2^; Continuous Measure)		
	(1)	(2)	(3)
	BMI_Diet	BMI_Individual	BMI_All Vars
MED_diet (follows = 1; no = 0)	−0.0033	0.0023	0.0106
	(0.0267)	(0.0353)	(0.0351)
female (female = 1; male = 0)		−0.5603 ***	−0.7368 ***
		(0.1567)	(0.1591)
marital status (NOT living with partner = 1; yes = 0)		−0.0481	−0.0864 *
		(0.0484)	(0.0469)
age		0.4092 ***	0.3826 ***
		(0.0416)	(0.0373)
age^2		−0.0031 ***	−0.0031 ***
		(0.0003)	(0.0003)
ethnicity (Hispanic = 1; Non-Hisp = 0)		0.3479 *	0.4085
		(0.1798)	(0.2531)
tertiary educ (tertiary = 1; No = 0)			−1.8495 ***
			(0.0909)
secondary educ (secondary = 1: No = 0)			−0.8852 ***
			(0.0743)
sports (active sports = 1; Not = 0)			−0.3072 ***
			(0.0282)
ever smoked daily (no = 1; yes = 0)			0.2074 ***
			(0.0658)
household income			−0.0000
			(0.0000)
household income^2			0.0000
			(0.0000)
employment status (employed = 1; no = 0)			−0.2747 ***
			(0.0440)
Constant	26.9819 ***	13.7261 ***	16.7651 ***
	(0.0258)	(1.4073)	(1.3087)
Observations	125,412	124,783	121,800
Number of id_n	84,317	83,826	82,361
Year FE	YES	YES	YES
COUNTRY FE	YES	YES	YES

Robust standard errors in parentheses. *** *p* < 0.01, ** *p* < 0.05, * *p* < 0.1.

**Table 4:** Regression analysis relating levels of depressive symptoms with the Mediterranean diet, individual and socio-demographic characteristics and lifestyle behaviors.

Covariates	Dependent Variable: EURO-D (Level of Depressive Symptoms Scale: 0 to 12)		
	(1)	(2)	(3)
	EURO-D_Diet	EURO-D_Individual	EURO-D_All Vars
MED_diet (follows = 1; no = 0)	−0.1490 **	−0.1366 **	−0.1118 **
	(0.0591)	(0.0534)	(0.0514)
female (female = 1; male = 0)		0.7515 ***	0.6932 ***
		(0.0484)	(0.0471)
marital status (NOT living with partner = 1; yes = 0)		0.4443 ***	0.3803 ***
		(0.0319)	(0.0300)
age		−0.2301 ***	−0.3010 ***
		(0.0136)	(0.0205)
age^2		0.0019 ***	0.0022 ***
		(0.0001)	(0.0002)
ethnicity (Hispanic = 1; Non-Hisp = 0)		0.3401 ***	0.2972 **
		(0.1040)	(0.1199)
tertiary educ (tertiary = 1; No = 0)			−0.6769 ***
			(0.0650)
secondary educ (secondary = 1: No = 0)			−0.4580 ***
			(0.0479)
sports (active sports = 1; Not = 0)			−0.5708 ***
			(0.0375)
ever smoked daily (no = 1; yes = 0)			−0.1936 ***
			(0.0168)
household income			−0.0000 **
			(0.0000)
household income^2			0.0000 ***
			(0.0000)
employment status (employed = 1; no = 0)			−0.4417 ***
			(0.0376)
Constant	1.9684 ***	8.2313 ***	12.4658 ***
	(0.0194)	(0.4836)	(0.6568)
Observations	126,018	125,045	121,803
Number of id_n	84,234	83,577	82,135
Year FE	YES	YES	YES
COUNTRY FE	YES	YES	YES

Robust standard errors in parentheses. *** *p* < 0.01, ** *p* < 0.05, * *p* < 0.1.

**Table 5:** Calibrating the Mediterranean diet variable for robustness.

Covariates	(1)	(2)	(3)	(4)	(5)	(6)
	CMD	CMD	BMI	BMI	EURO-D	EURO-D
MED2 (follows = 1; no = 0)	−0.0229 ***	−0.0174 **	0.0111	0.0210	−0.2014 ***	−0.1510 ***
	(0.0088)	(0.0088)	(0.0215)	(0.0285)	(0.0408)	(0.0368)
female (female = 1; male = 0)		−0.0859 ***		−0.7370 ***		0.6952 ***
		(0.0228)		(0.1591)		(0.0466)
marital status (NOT living with partner = 1; yes = 0)		−0.0053		−0.0855 *		0.3744 ***
		(0.0086)		(0.0464)		(0.0306)
age		0.0513 ***		0.3823 ***		−0.2995 ***
		(0.0084)		(0.0373)		(0.0207)
age^2		−0.0002 ***		−0.0031***		0.0022 ***
		(0.0001)		(0.0003)		(0.0002)
ethnicity (Hispanic = 1; Non-Hisp = 0)		0.1010 **		0.4078		0.3020 **
		(0.0504)		(0.2523)		(0.1200)
tertiary educ (tertiary = 1; No = 0)		−0.2211 ***		−1.8502 ***		−0.6720 ***
		(0.0220)		(0.0910)		(0.0652)
secondary educ (secondary = 1: No = 0)		−0.1196 ***		−0.8854 ***		−0.4562 ***
		(0.0211)		(0.0745)		(0.0480)
sports (active sports = 1; Not = 0)		−0.1713 ***		−0.3078 ***		−0.5660 ***
		(0.0177)		(0.0280)		(0.0369)
ever smoked daily (no = 1; yes = 0)		−0.0536 ***		0.2072 ***		−0.1923 ***
		(0.0126)		(0.0659)		(0.0167)
household income		−0.0000		−0.0000		−0.0000 **
		(0.0000)		(0.0000)		(0.0000)
household income^2		0.0000		0.0000		0.0000 **
		(0.0000)		(0.0000)		(0.0000)
employment status (employed = 1; no = 0)		−0.1303 ***		−0.2748 ***		−0.4412 ***
		(0.0129)		(0.0440)		(0.0375)
Constant	0.8500 ***	−1.1873 ***	26.9788 ***	16.7752 ***	1.9911 ***	12.4211 ***
	(0.0061)	(0.2965)	(0.0251)	(1.3072)	(0.0207)	(0.6645)
Observations	129,083	124,549	125,412	121,800	126,018	121,803
Number of id_n	85,836	83,600	84,317	82,361	84,234	82,135
Year FE	YES	YES	YES	YES	YES	YES
COUNTRY FE	YES	YES	YES	YES	YES	YES

Robust standard errors in parentheses. *** *p* < 0.01, ** *p* < 0.05, * *p* < 0.1.

## Appendix A

**Table A1:** CMD (frequency and percentages).

Incidence of CMD	Freq.	Percent
0	99,561	44.37
1	69,426	30.94
2	37,112	16.54
3	14,588	6.5
4	3292	1.47
5	385	0.17
Total	224,364	

**Table A2:** BMI (frequency and percentages).

BMI (kg/m^2^)	Freq.	%
Below 18.5—underweight	2350	1.08
18.5−24.9—normal	74,574	34.18
25−29.9—overweight	90,671	41.56
30 and above—obese	50,556	23.17
Total	218,151	

**Table A3:** Depressive symptoms (frequency and percentages).

EURO-D Scale		
Depressive Symptoms	Freq.	%
0. None	37,553	22.39
1	36,280	21.63
2	28,622	17.07
3	21,816	13.01
4	15,741	9.39
5	10,697	6.38
6	7054	4.21
7	4452	2.65
8	2716	1.62
9	1567	0.93
10	814	0.49
11	309	0.18
12	77	0.05
Total	167,698	

## Appendix B

**Table A4:** Dietary patterns and number of respondents, with percentages.

**How Often Serving of Legumes or Eggs (Br027_Inv)**		**%**
5—Every day	13,585	10.72
4—3−6 times week	34,249	27.03
3—Twice week	36,680	28.95
2—Once week	29,161	23.02
1—Less than once a week	13,013	10.27
Total	126,688	
**How often serving of meat, fish or chicken (br028_inv)**		**%**
5—Every day	49,449	39.02
4—3−6 times week	53,076	41.88
3—Twice week	16,536	13.05
2—Once week	5585	4.41
1—Less than once a week	2076	1.64
Total	126,722	
**How often serving of fruits or vegetables (br029_inv)**		**%**
5—Every day	97,848	77.21
4—3−6 times week	20,140	15.89
3—Twice week	5347	4.22
2—Once week	2013	1.59
1—Less than once a week	1381	1.09
Total	126,729	

## Appendix C

**Table A5:** Country groupings based on the UN geoscheme.

**Eastern Europe**	**Western Europe**
Bulgaria	Austria
Czech Republic	Belgium
Hungary	France
Poland	Germany
Romania	Luxembourg
Slovakia	Netherlands
	Switzerland
**Northern Europe**	**Southern Europe**
Denmark	Croatia
Estonia	Cyprus
Finland	Greece
Latvia	Italy
Lithuania	Malta
Sweden	Portugal
	Slovenia
	Spain
